# Pathogenic sphingosine 1-phosphate pathway in psoriasis: a critical review of its pathogenic significance and potential as a therapeutic target

**DOI:** 10.1186/s12944-023-01813-3

**Published:** 2023-04-18

**Authors:** Yuechun Zhao, Yuheng Zhang, Jiaqi Li, Ningxin Zhang, Qiubai Jin, Yuxia Qi, Ping Song

**Affiliations:** 1 Guang’anmen Hospital China Academy of Chinese Medical Sciences, Beijing, China; 2 Beijing University of Traditional Chinese Medicine, Chaoyang, China

**Keywords:** Psoriasis, Sphingosine 1-phosphate, Immunity, Small molecule drugs

## Abstract

Sphingosine-1-phosphate (S1P) is a sphingolipid mediator that exerts a variety of biological functions, including immune, cardiovascular, and neurological regulation as well as tumor promotion, through high-affinity G protein-coupled receptors (S1P_1-5_). It has been reported that circulating S1P levels remain higher in patients with psoriasis than in healthy individuals and that circulating S1P levels do not decrease after anti-TNF-α treatment in those patients. The S1P-S1PR signaling system plays an important role in inhibiting keratinocyte proliferation, regulating lymphocyte migration, and promoting angiogenesis, thus contributing to the regulation of psoriasis pathogenesis. Here, we review the mechanisms by which S1P-S1PR signaling affects the development of psoriasis and the available clinical/preclinical evidence for targeting S1P-S1PR in psoriasis. S1P-S1PR signaling mechanisms may partially explain the link between psoriasis and its comorbidities. Although the detailed mechanisms remain to be elucidated, S1P may be a new target for future psoriasis remission.

## Introduction

Psoriasis is a common disease that is complex, chronic, and immune-mediated and involves the skin and joints, with a prevalence ranging from 0.09% to 11.43% in different countries, and approximately 100 million adults suffer from the disease [1], which severely affects their quality of life. It is currently believed that psoriasis is influenced by a variety of factors, such as genetics, infection, medication and lifestyle (smoking, obesity and alcohol consumption) [2, 3]. Research on the pathogenesis of psoriasis has developed rapidly over the past 15 years, involving multiple intracellular signaling pathways (PI3K, AKT, JAK-STAT, JNK, WNT, etc.), and the IL-23/Th17 axis is now recognized as a key link in the immunopathogenesis of psoriasis. As research on the pathogenesis of psoriasis progresses, more biological targets have been identified [4–6]. The systemic inflammation caused by psoriasis promotes the development of comorbidities, with 73% of patients suffering from at least one comorbidity [7]. The common comorbidities are psoriatic arthritis, Crohn's disease, metabolic syndrome, nonalcoholic fatty liver disease, and cardiovascular disease. Therefore, finding pathomechanical associations between psoriasis and its comorbidities can be a boon for the majority of psoriasis patients (especially those with higher severity).

Sphingosine 1-phosphate (S1P) is a sphingolipid mediator that regulates various biological processes in health and disease through the high-affinity G protein-coupled receptors S1P_1-5_. S1P-S1PR have been found to be involved in the pathogenesis of several immune diseases, such as multiple atherosclerosis (MS), obstructive coronary artery disease, inflammatory bowel disease (IBD), tumors, and psoriasis [8–12]. In addition, S1P and its signaling have been reported to regulate angiogenesis, modulate lymphocyte excretion, regulate the migration of various immune cells, including Th17/Treg cells, and affect IL-23-mediated signaling [13]. S1P and its analogs (represented by FTY720) have been shown to inhibit the proliferation of keratinocytes. Antonio Checa et al. [14] found that circulating levels of S1P remained high after treatment with biologic agents, suggesting that increased levels of S1P may be the cause of psoriasis relapse. Moreover, circulating S1P levels were higher in psoriasis patients than in healthy subjects and correlated with body weight, and Kozłowska et al. found [15] that S1P was a marker of insulin resistance and obesity. All evidence suggests that S1P and its signaling may be a link between psoriasis and metabolic-related diseases (obesity, metabolic syndrome, cardiovascular disease) and that targeting S1P may offer new opportunities for the treatment of psoriasis. This review highlights the importance of the S1P-S1PR signaling system in the inflammatory process, describes the specific mechanisms by which S1P and its signaling are involved in the pathogenesis of psoriasis, describes the available clinical/preclinical evidence for targeting S1P-S1PR in psoriasis, and identifies S1PR modifiers (e.g., S1P-S1PR). Ponesimod and Syl930) and their potential targets.

## The Source and Function of S1P

Sphingolipids are prevalent components of eukaryotic cell membranes, and sphingosin-1-phosphate (S1P) is a bioactive sphingolipid metabolite that works in an autocrine or paracrine manner [16]. Its source is ceramide (CER), which consists of a sphingosine group and a variable-length amide-linked acyl chain that is converted intracellularly by deacylation to sphingosine and carboxylate. Then, sphingosine is phosphorylated by sphingosine kinase 1 (SPHK1) or sphingosine kinase 2 (SPHK2) to produce S1P in the lysosome and endoplasmic reticulum [17]. Its intracellular level is regulated by S1P biosynthesis and the S1P degradation pathway. It has the capability to act both as an intracellular second messenger and as a ligand for G protein-coupled receptors (GPCRs), regulating different physiological processes by binding to five specific receptors, S1P_1-5_. Types 1, 2 and 3 are predominantly expressed in cardiovascular tissues, and the SPHK/S1P axis is involved in the development and function of the cardiovascular system. The function includes the regulation of heart rate, cardiac contractility and vascular tone [18]. It has been proven that S1P is associated with improved poor cardiac remodeling after myocardial infarction [19]. Byambasuren Ganbaatar et al. [20] found that S1P_2_ antagonists reduced endothelial dysfunction and prevented atherogenesis. S1PR4 plays a role in the migration and differentiation of immune cells, while S1PR5 plays a role in the immune and nervous systems. S1PR5 can also help NK cells translocate into tissues and control brain endothelial barrier function and permeability [21] (Table 1).

**Table 1:**
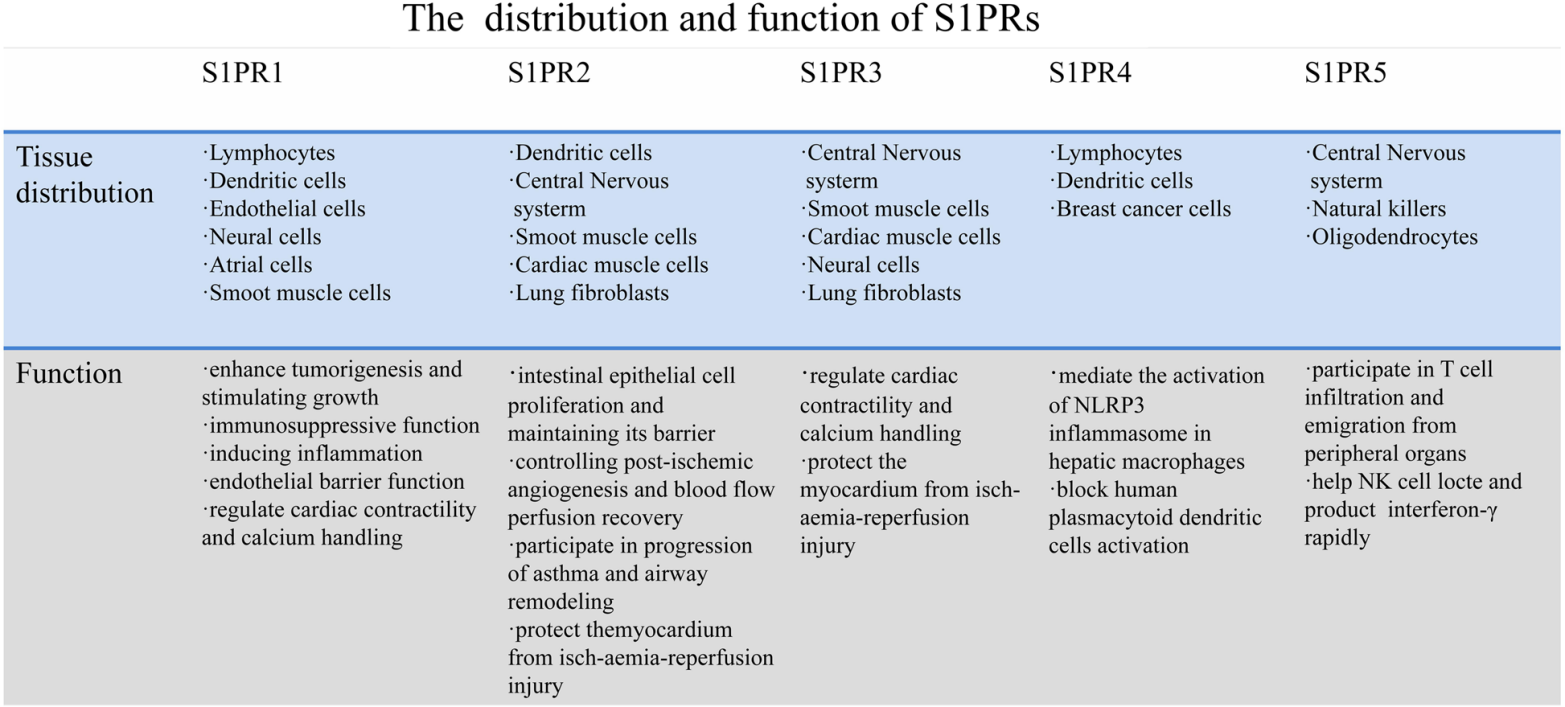
The distribution and function of S1PRS

S1P occupies an important position in the immune system. In general, S1P stimulation increases the permeability of lymphatic endothelial cells, upregulates the expression of T-cell and interendothelial cell adhesion proteins, and regulates the migration of CD4 + T cells to the initial lymphatic vessels. Chemokines CCL21 and S1P released from lymphatic endothelial cells regulate lymph node migration of T cells to reach the site of inflammation and participate in the immune response. In summary, S1P is involved in the development, maturation and migration of T cells in immune organs. It initiates translymphatic endothelial migration (TEM) by influencing the interaction between lymphatic endothelial cells and T cells, thus causing T cells to migrate out of lymph nodes. Disruption of the S1P concentration gradient in the thymus, where T cells mature, results in impaired thymic migration of mature T cells. In the spleen, S1P also promotes lymphocyte emigration and recirculation from the white pulp to the red pulp.

**Fig. 1:**
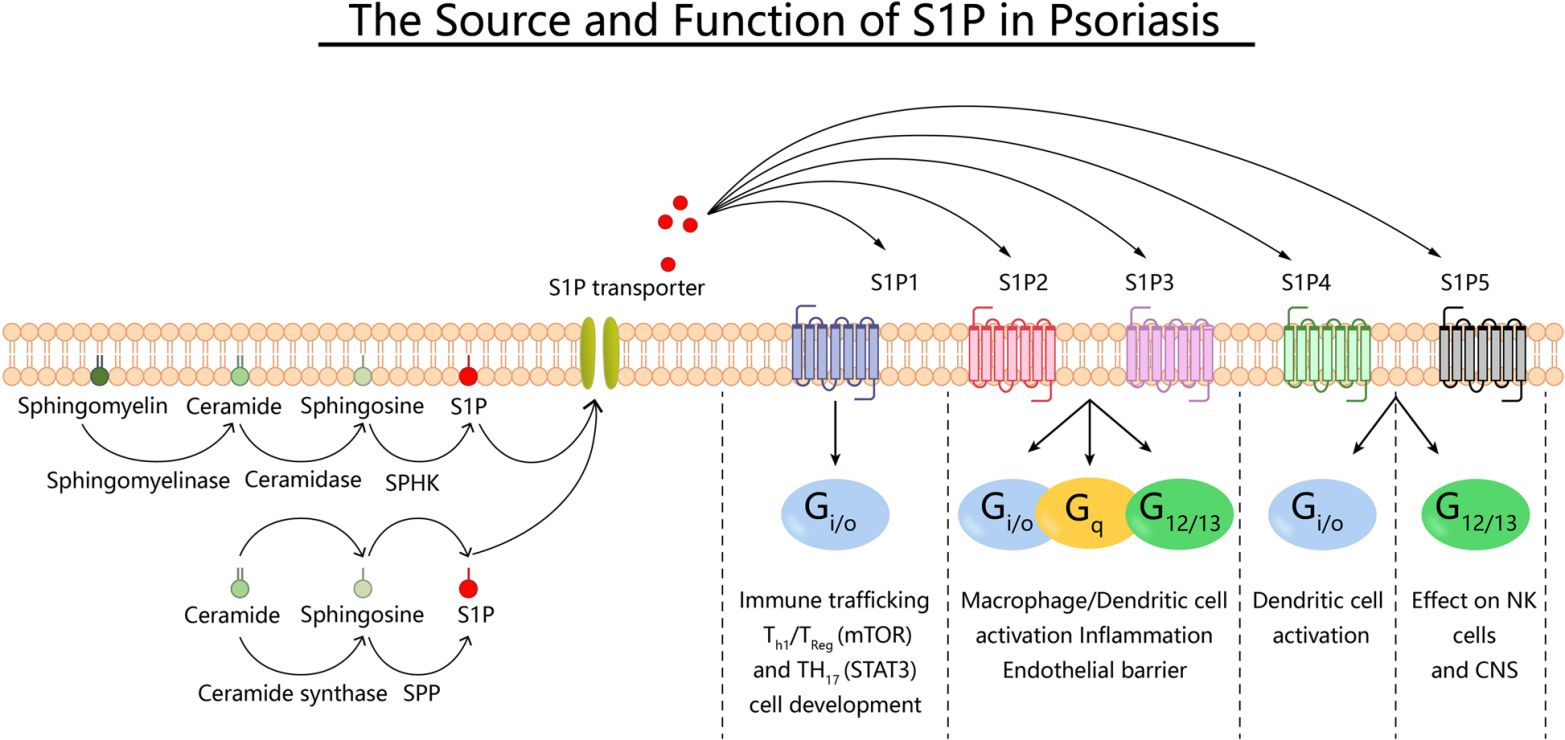
The Source and Function of S1P in Psoriasis

S1P signaling, via GPCRs, affects the entire mammalian physiology, from the immune system to the nervous system and the circulation of skeletal muscle organs. It also plays a significant role in the development of psoriasis. Psoriasis is a chronic inflammatory and proliferative disease caused by genetic and environmental factors and is characterized by excessive keratinocyte proliferation, increased dermal angiogenesis, and immune cell infiltration, with localized skin erythema, scaling, and pruritus. In patients with psoriasis, serum concentrations of CER are significantly lower and S1P concentrations are significantly higher. S1P is an important factor in the proliferation and differentiation of human keratinocytes, and Suwon Jeon et al. demonstrated that human epidermal keratinocyte differentiation is associated with elevated S1P and downregulation of S1P lyase [22]. It also causes excitation of nociceptive receptors and persistent pain in mice and humans through activation of S1PR3 and S1PR1 [23]. When S1P was injected intradermally into mice, S1PR3 in pruritic c-fiber neurons was stimulated, causing itching and pain. There is ample evidence that the skin inflammation exhibited by psoriasis is caused by inflammatory mediators such as TNF-α, that macrophages are a key source of TNF-α and that S1P has a significant effect on this process by activating S1PR1 on macrophages [24]. Interleukin-1β (IL-1β), also derived from macrophages, promotes vascular and lymphatic vasculature production, and S1PR1, when combined with S1P, activates STAT3 phosphorylation and promotes the development of chronic inflammation. Numerous studies have suggested that S1P plays an important role in the pathogenesis of psoriasis through direct or indirect actions (Fig. 1).

## S1P and its signaling affect psoriasis pathogenesis

### S1P inhibits the proliferation of keratinocytes and induces their differentiation

Psoriasis is a chronic inflammatory skin disease characterized by abnormal proliferation/differentiation of keratinocytes and excessive infiltration of immune cells in the dermis and epidermis. In particular, keratinocytes not only act as "signal transducers" to trigger epidermal inflammation in psoriasis but also as executors of the immune pathway in psoriasis [25]. It has also been recently shown that basal keratinocytes of stem cells have a similar ability to acquire long-term memory of inflammation and may regulate psoriasis relapse [26]. Therefore, keratinocytes have been used as a potential strategy for psoriasis treatment. In recent years, we found that S1P and its signaling inhibited the proliferation of keratinocytes and induced their differentiation [27]. Therefore, targeted therapeutic strategies for S1P and its signaling could alleviate psoriasis symptoms and reduce its recurrence by inhibiting keratinocyte proliferation, perhaps with fewer side effects and lower posttreatment recurrence rates than current biologic agents.

S1P acts as an intracellular second messenger by coupling to the G protein-coupled receptors S1P_1-5_ to trigger a range of biological effects, including cell proliferation, immunosuppression and cardiovascular function. S1P inhibits keratinocyte proliferation by coupling to S1P_2_, with no cytotoxicity. Among them, S1PR2 is mainly involved in inhibiting keratinocyte growth through activation of protein kinase C (PKC) and subsequent Akt dephosphorylation. Kim et al. [28] showed that although S1P is known as a mitogenic signaling molecule, S1P can play an important role in the negative regulation of keratinocyte proliferation through prolonged activation of ERK and cell cycle arrest induced by transient inactivation of Akt/PKB, and this inhibitory effect can be significantly reversed by pertussis toxin (PTX).

Jeon et al. [22] found that inhibition of S1P lyase elevated S1P levels and induced G1 phase growth arrest and keratinocyte differentiation through inhibition of cyclin D1, cyclin D3, and the cyclin-dependent kinases CDK2, CDK4, and CDK6. This suggests that S1P lyase may be a regulatory point for human keratinocyte proliferation.

S1P not only regulates the proliferation of human keratinocytes but also transiently increases the concentration of free Ca^2+^ in these cells. This process is mediated by stimulation of phospholipase C and involves the mobilization of Ca^2+^ from thapsigargin-sensitive stores and subsequent Ca^2+^ influx. Ca^2+^ is the most critical signal in the differentiation process of keratinocytes, prompting their transformation into keratinocytes. S1P involvement in intracellular Ca2 + level elevation can promote this differentiation process. Lichte et al. [29] demonstrated that the S1P-induced Ca^2+^ increase in keratinocytes was mediated by S1PR3.

S1P protects keratinocytes from apoptosis. Moriue et al. [30] found that S1P attenuates H2O2-induced apoptosis in HaCaT cells through phosphorylation of Akt. Previously, similar reports have been made, for example, that S1P prevents apoptosis in keratinocyte cultures in response to stimuli such as TNF-α and UVB [31, 32]. Notably, Elisabeth I. Schmitz et al. also found that S1P protects human keratinocytes from apoptosis by activating the S1PR3 subtype to produce protective amounts of NO [33].

In addition to its proliferative, differentiation and anti-apoptotic effects on keratinocytes, S1P is also engaged in immune responses and inflammatory mechanisms. Oizumi et al. demonstrated that ceramidase induces inflammatory mediators, including endothelin-1, TNF-α and IL-8, in human keratinocytes via S1P [34]. It is well known that endothelin-1 causes nonhistamine-dependent pruritus in humans [35] and that pruritus is an essential indication of psoriasis severity. S1P produced by keratinocytes is involved in endothelin-1-mediated pruritus in the pathogenesis of psoriasis. TNF-α inhibitors have been widely used in various psoriatic diseases and are reasonable targets for the treatment of chronic psoriasis [36]. S1P may primarily induce TNF-α production through S1P_1_ and/or S1P_3_ in keratinocytes. Igawa et al. found that S1P increased the expression of IL-36γ, TNF-α and IL-8 in normal human epidermal keratinocytes (NHEKs) in vitro and that this expression was tightly controlled by S1PR1/2 in keratinocytes [37]. This allows S1P to have completely opposite effects on the local skin of psoriasis. On the one hand, S1P reduces skin pathology by inhibiting keratinocyte proliferation and inducing keratinocyte differentiation; on the other hand, S1P causes keratinocytes to produce metabolites that act as stimuli for inflammatory immune responses to aggravate skin lesions.

### S1P and its signal transduction regulate the immune process in psoriasis

Immune responses occupy an important place in the pathogenesis of psoriasis, especially the IL-23/IL-17 axis, which depends on the function of Th17 cells. Immune-related cells such as dendritic cells (DCs) and macrophages, as well as Toll-like receptors and cytokines such as interferon (IFN)- α, TNF-α, IFN-ɤ, IL-12, IL-22, IL-23, and IL-17, are closely associated with psoriasis pathogenesis. S1P is involved in a key immune link in psoriasis pathogenesis and can be considered a circulating marker. On the one hand, S1P helps lymphocytes to exclude from lymph nodes by binding to S1P_1-5_ and reach the site of inflammation [38]. On the other hand, it regulates the activation and polarization of several types of immune cells (including Th17 cells) [39].

S1P is enriched in lymph and blood but present in small amounts in the intracellular and interstitial fluid, forming a steep S1P gradient. This gradient controls the efflux of lymphocytes, which are then carried into the circulation by various carriers, such as Mfsd2b and Spns2 [40, 41]. Psoriasis is a disease mediated by T lymphocytes and is characterized by infiltration of a large number of immune cells (lymphocytes, macrophages and neutrophils). This persistent inflammation will aggravate the formation of plaques, while cytokines produced by immune-related cells such as IL-17 and TNF-α will further recruit immune cells and form a vicious circle of local inflammation. If there is no S1P_1_ on T/B cells, then they cannot emerge from the secondary lymphoid organs, and the number of peripheral local lymphocytes will be relatively reduced. Inhibitors of S1P lyase (S1PL) or S1P antagonists such as FTY720 (fingolimod) prevent lymphocyte migration from lymphoid organs, leading to lymphocyte decrease [42]. Therefore, targeting the S1P signaling axis can alleviate psoriasis by inhibiting the efflux of key immune cells, which has been widely clinically proven.

Th17 cells are known to have a vital function in the pathogenesis of psoriasis and are key driver cells in the development of psoriatic inflammation. It has been indicated that circulating S1P levels are elevated in patients with psoriasis compared to healthy patients. It has been shown that S1P can enhance the development of Th17 cells and locally block the production of S1P, which can reduce serum levels of IL-17A. S1P analogs (SEW2871, BAF312 and FTY720) directly or indirectly inhibit Th17 cell differentiation in vitro through their negative effects on antigen-presenting cells and reduce the production of the proinflammatory cytokines IL-23, IL-6, and IL-1β while increasing Treg differentiation of primitive CD4 + T cells. On the other hand, these three S1P analogs reduced the phosphorylation response of STAT4, NF-κB and AKT in response to IL-23, demonstrating that the S1P signaling pathway crosses over with IL-23 signaling to further limit the pathogenicity and expansion of Th17 [43]. All evidence indicates that blocking S1P and its signaling can affect the activation and polarization of Th17 cells, thus alleviating the inflammatory response in psoriasis. Sun-Hye SHIN et al. [44] found different effects of SPHK1 and SPHK2 on Th17 cells, and only inhibition of SPHK2 directly affected Th17 differentiation of naïve CD4 + T cells. S1P lyase, encoded by the Sgpl1 gene, can degrade S1P. Yang et al. [45] demonstrated for the first time the formation of hyperpigmented patches at skin lesions in an Sgpl1-deficient mouse model. The presence of an enrichment of IL17a-producing Vγ6 + cells in the skin of SGPL1-deficient patients exhibited hyperchromatic patches that could reflect their biased IL17-related proinflammatory response in the cutaneous immune system.

Dendritic cells (DCs) are key players in the immune response to psoriasis, producing mainly IL-12/IL-23, which is essential for the development and maintenance of psoriasis. Dillmann et al. [46] showed that S1P secreted by plasmacytoid dendritic cells (pDCs) significantly reduces IFN-α production by coupling with S1P_4_, thereby shifting cytokine production from Th1 (IFN-g) to regulatory (IL-10) in T cells. This is tantamount to providing evidence for S1PR4 agonists in the treatment of pathogenic IFN-α production. In addition, Schuster et al. [47] found that although the deletion of S1PR4 will not reduce the production of IL-17 in IMQ psoriasis model mice, it can reduce CCL2, IL-6 and CXCL1 (an important vector of psoriasis immune response), also confirming that S1PR4 may be a target for reducing permanent inflammatory responses. Prior to this, Schaper et al. [48] investigated the role of S1P on cytokines in DCs and showed that S1P has an anti-inflammatory effect on the production of IL-12 family cytokines (IL-12p70, IL-23 and IL-27). In addition, Langerhans cells (LCs) are specialized dendritic cells located in the epidermis that are the initiators of the T-cell immune response, and the S1P_1_ and S1P_3_ subtypes are responsible for the migratory response of LCs [49]. S1P applied topically regulates the inhibition of antigen uptake by LCs through the ABCC1/S1P_2_ and PI3K axes to eliminate local inflammation in the skin [50]. Bock et al. [51] reported that S1P regulates the maturation of human LCs, including key aspects of cytokine release and migration, and is also closely associated with skin surface inflammation.

Macrophages are a key source of TNF-α and express various S1PRs. Macrophages have also been shown to induce psoriasis in the absence of T and B cells, and Treg cells can inhibit this proinflammatory activity of macrophages [52]. Interestingly, Syed et al. [24] found that bone marrow S1PR1 exhibited anti-inflammatory effects in early psoriasis-like skin inflammation in mice, which is completely opposite to the expected results, suggesting that there is a more complex compensatory mechanism involved. In addition, myeloid-specific S1PR1 deletion mice showed decreased lymphocytes and increased vascularity, whereas previous reports pointed to neovascularization as an exacerbating factor of psoriatic inflammation, revealing a different role of macrophage S1P-S1PR1 signaling in lymphogenesis and angiogenesis [24]. Furthermore, myeloid S1PR1 signaling was also reported to induce IL-1β production by enhancing macrophage NLRP3 inflammatory vesicle activity, and SPHK is involved in this process [53].

Mast cells are widely present in psoriatic lesions and are overactivated [54]. Mast cells play a regulatory immune function by releasing a variety of inflammatory mediators and form a complex mast cell-centered immune network with T cells, Tregs, and keratinocytes [55] In addition, mast cells express two isoforms of SPHK (SPHK1 and SPHK2), which are activated upon FcεRI stimulation and produce higher levels of S1P [56]. Moreover, mast cells also express S1PRs, in which the activation of S1PR1 promotes mast cell migration toward antigens and the activation of S1PR2 triggers mast cell degranulation. These processes aggravate the occurrence of inflammation [57]. Thus, there is strong signaling crosstalk between mast cells and the S1P axis, and targeting the S1P axis to regulate mast cells is also crucial evidence that S1P treatment improves inflammation in psoriasis. Megan et al. [58] found that an SPHK1 inhibitor attenuates mast cell-mediated allergic responses and inhibits NF-κB activation, which is widely reported in many pulmonary diseases, such as allergic rhinitis and asthma. It has also been proven in inflammatory skin diseases. Park et al. reported that blockade of S1PR2 attenuated mast cell aggregation and reduced inflammatory cytokine levels (IL-4, IL-13, IL-17 and IFN-γ) in mice with atopic dermatitis [59].

### S1P induces dermal angiogenesis in psoriasis

The SPHK/S1P/S1PR signaling pathway is associated with multiple physiopathological processes in psoriasis; however, its relationship with increased dermal angiogenesis in psoriasis is not completely clear. Angiogenesis is considered to be one of the principal events in the development of psoriasis, and its histological features include an increased distribution of dermal blood vessels. The maintenance of inflammation in psoriasis is also dependent on the close interaction of inflammatory Th1/Th17 cells with the vascular bed, facilitating their transfer from the periphery to the dermis [60]. Sites of inflammation are often accompanied by increased vascular permeability to help immune cells reach the site of inflammation to eliminate pathogens. However, increased vascular permeability can further exacerbate the inflammatory response. Thus, angiogenesis is not only a cofactor but also an inducer of psoriasis development. Vascular endothelial growth factor, hypoxia-inducible factor, tumor necrosis factor, IL-8 and angiopoietin are generally considered to be the main players in psoriasis angiogenesis [60]. However, in the last 20 years, it has been observed that S1P and its signaling also have an important role in the development and maturation of blood vessels.

**Fig. 2:**
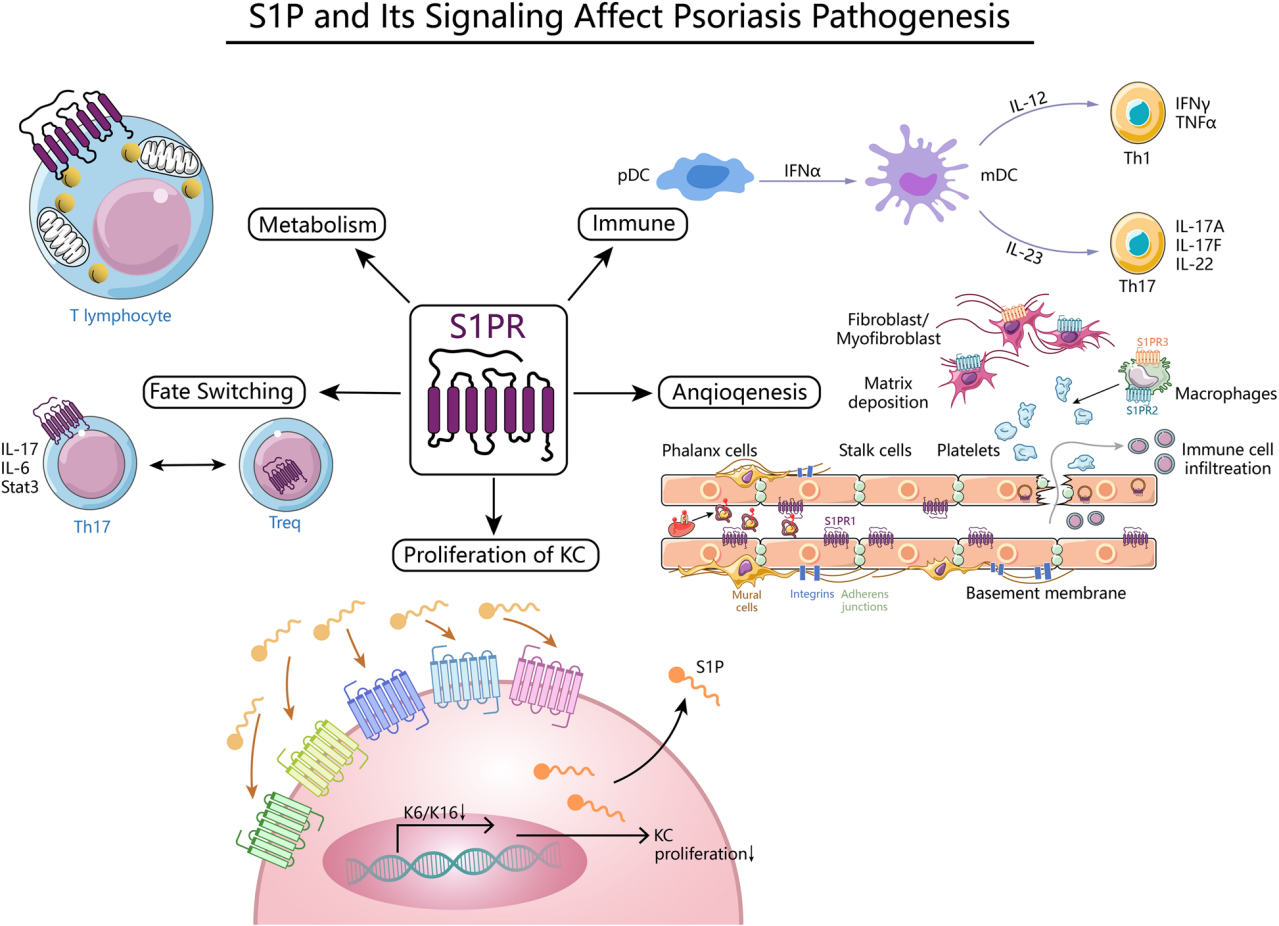
S1P and Its Signaling Affect Psoriasis Pathogenesis

Lee et al. [61] first reported that S1P stimulates angiogenesis in mice via S1PR1 and S1PR3. Subsequently, Du et al. [62] proved that inflammatory mediators such as LPS and TNF-α induced S1PR2 expression in the endothelium and that upregulation of S1PR2 might be in charge of the impaired barrier function of endothelial cells. Thus, increased angiogenesis and disruption of vascular barrier function in the dermis were mainly associated with S1PR1, S1PR2, and S1PR3 expressed by endothelial cells [63]. S1P and its signaling role in angiogenesis were subsequently introduced to a variety of diseases, such as cancer, cardiovascular disease, and inflammatory bowel disease [64–66]. Recently, some progress has been made in the study of this role in skin wound healing, inflammatory skin diseases (e.g., psoriasis and atopic dermatitis.) Aoki et al. [67] found that S1P was related to the increase in angiogenesis and the recruitment of inflammatory cells by regulating the loss and overexpression of SPHK1 in the local wounds of mice. Therefore, the local topical application of S1P can promote early wound healing and later scar repair. Yoon et al. [68] reduced angiogenesis in a mouse model of atopic dermatitis using a novel antagonist of S1PR1, which effectively alleviated atopic dermatitis symptoms in vivo/in vitro. Syed et al. [24] used imiquimod (IMQ) to induce psoriasis-like dermatitis in a mouse model of myeloid-specific S1PR1 deletion to explore lymphatic versus angiogenesis in conditional knockout mice and found that increased inflammation in mice was associated with increased vascularity but decreased lymphatic vessel density. In summary, the SPHK/S1P/S1PR signaling axis is one of the multiple mechanisms of increased dermal angiogenesis in psoriasis, and the disruption of the angiogenic system caused by S1P may be an important mediator of the link between psoriasis and multiple comorbidities (especially cardiovascular disease). Therefore, S1P-based therapy provides an important mechanism for the development of early psoriasis, and targeted S1P therapy provides new therapeutic ideas for the occurrence of early psoriasis, maintenance of psoriasis in the later stage, and combination with other diseases (Fig. 2).

## S1P and S1PR modulators for the treatment of psoriasis

The immunopathogenesis of psoriasis, an inflammatory skin disease mediated by immune cells, is now becoming clear, and biologics developed for its key immune aspects, such as tumor necrosis factor (TNF)-α inhibitors (infliximab, adalimumab, polyethylene glycol certolizumab), IL-23 inhibitors (utekizumab, guselumab, tildrakizumab, risankizumab) and IL-17 inhibitors (suginumab, ixekizumab, brodalumab), have also achieved some validation of clinical effects. However, there is still some relapse rate with biologic therapy, especially in patients with more severe psoriasis, and Checa et al. [14] found that although biologics improved psoriatic skin symptoms, they did not reduce circulating S1P levels, so that still high S1P levels may be a key cause of psoriasis relapse. Psoriasis is closely related to metabolic syndrome, in which both lipids and lipid metabolism have significant effects [15]. Myśliwiec et al. [69] also suggested that S1P is an intermediate link between psoriasis and multiple metabolic-related diseases. Since S1P signaling is involved in multiple immune functions, targeted S1P therapeutics have been clinically validated for a variety of immune diseases, such as nonalcoholic fatty liver disease (NAFLD), multiple sclerosis (MS), ischemic stroke, and coronary artery disease [70–73]. In the last decade, the role of S1P and its signaling in recalcitrant skin diseases has also been elucidated. S1P is involved in the development of psoriasis through several links, and therefore, targeting S1P therapy is indispensable for establishing a more complete treatment strategy for psoriasis (Table 2).

**Table 2:**
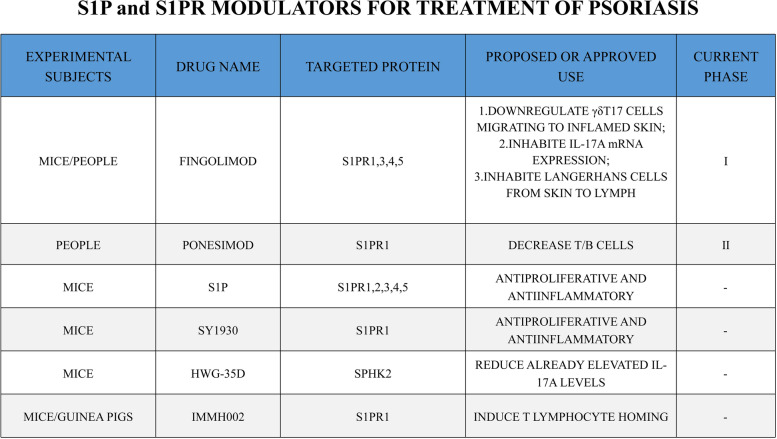
S1P and S1PR modulators for treatment of psoriasis

Fingolimod is the first approved oral immunosuppressant that targets all S1PRs except S1PR2 as a nonselective agonist, exerting immunosuppressive effects by inducing S1PR internalization/degradation and inhibiting lymphocyte efflux. Initially, used as a treatment for multiple sclerosis with significant clinical efficacy, researchers are evaluating the potential and potential risks of fingolimod in other immune diseases, including psoriasis [74]. Ramírez-Valle et al. [75] found that γδT17 cells migrate to inflamed skin in an S1PR1-dependent manner and that fingolimod downregulates this response. Similarly, Okura et al. [76] investigated the effect of fingolimod on psoriasis in mice and found that fingolimod inhibited the mRNA expression level of IL-17A in skin lesions and inhibited the efflux of Langerhans cells from the skin to the lymph nodes, thereby reducing psoriatic skin lesions in mice, which is consistent with previously reported results.

Ponesimod/ACT-128800 is a selective modulator of S1PR1. Although several S1P receptor modulators are in clinical development, ponesimod remains the only S1P receptor modulator studied in patients with psoriasis. In a phase 2 clinical trial, ponesimod demonstrated positive effects in 77.4% of patients with psoriasis [77]. The main mechanism of ponesimod in the treatment of psoriasis is that psoriasis is a T-cell-mediated inflammatory skin disease, and ponesimod induces a rapid, dose dependent, and reversible reduction in peripheral blood lymphocyte count by blocking the egress of lymphocytes from lymphoid organs. Reducing the entry of pathogenic T cells into the skin may be a way to treat this disease. However, systemic side effects such as lymphopenia and transient bradycardia were reported, so clinical validation of ponesimod did not proceed to phase 3 due to safety concerns. Daniele D'Ambrosio et al. [78] found that administration of different doses of ponesimod to mice resulted in a rapid and transient reduction in the number of circulating T and B cells (maximum decrease of 67% to 89%), with the most pronounced reduction in pro-inflammatory CD4 + T cells (e.g., Th1/Th17) at doses of 20–75 mg, an effect achieved mainly by isolated lymphocytes. In addition, it has been proven that the local application of S1P receptor modulators is effective in several mouse dermatitis models [79–81], and the development of local preparations of S1P receptor modulators has made remarkable progress. Bell et al. [82] developed a topical external soft drug for local external use of an S1PR1 agonist by modifying ponesimod to reduce the side effects of systemic administration, providing an important reference for further optimization of ponesimod in the treatment of psoriasis.

In addition, other targeted therapeutic agents targeting the S1P axis have been reported to have therapeutic potential for psoriasis. Schaper et al. [83] found that S1P reduced imiquimod-induced ear skin epidermal hyperproliferation and significantly inhibited ear swelling, showing antiproliferative and anti-inflammatory effects in psoriatic mice, while the S1P analog fingolimod showed no significant effect. MingJi et al. [84] tested a newly developed selective S1P_1_ receptor modulator, Syl930, and showed strong antiproliferative and anti-inflammatory effects of orally administered Syl930, specifically in terms of reducing pathological skin thickening, inhibiting basal cell proliferation, and increasing granular layer scales in the mouse tail test. IMMH002 is a novel orally active S1P_1_ modulator, and JingJin et al. [85] Using different animal models, IMMH002 was shown to significantly alleviate psoriasis PASI scores and pathological damage by inducing T lymphocyte homing, thereby reducing T lymphocyte allocation in the skin periphery versus the blood. Shin et al. [86] used a novel SPHK2 inhibitor, HWG-35D, applied topically to improve IMQ-induced localized psoriatic lesions in mice and to reduce already elevated IL-17A levels.

## Conclusions and future perspectives

S1P and its signaling play an important role in the pathogenesis of many autoimmune diseases, and it is intimately involved in psoriasis pathogenesis by inhibiting keratinocyte proliferation, regulating immune cell migration, and promoting dermal angiogenesis and repair in several ways. Additionally, S1P has been found to be a potential link between psoriasis and its comorbidities as a biomarker for various metabolic diseases, such as diabetes and cardiovascular disease. Topical application of S1P and its analogs as well as oral S1P receptor inhibitors have been effective in preclinical trials and clinical trials in psoriasis, but adverse effects have been reported with oral S1P receptor inhibitors due to their broad action. Evidence has accumulated in recent years that S1P and its signaling are effective pharmacological targets for immune diseases, particularly in the treatment of T-cell driven inflammatory skin diseases represented by psoriasis. Clinical trials of ponesimod for psoriasis have demonstrated the effectiveness of S1P-based therapy, with the advantage of effectiveness in lower doses. Notably, significant progress has followed in the development of topical application of S1P and S1P receptor modulators, and topical application is expected to avoid the majority of S1P receptor modulator class side effects. Topical application of S1P receptor modulators (FTY720) combined with calcium-regulated neurophosphatase inhibitors has been reported to be the most effective in reversing local skin inflammation with negligible side effects [87], promising to be a candidate for steroid-resistant dermatitis. However, most of the evidence is based on animal trials, and proof of principle in human patients remains inadequate. Therefore, more topical agents targeting the S1P axis are in progress, while refining the targeting of S1P receptor inhibitors is a potential direction for future research into immunotherapeutic approaches for psoriasis.

## Data Availability

Not applicable.
